# Phytochemicals as Immunomodulatory Molecules in Cancer Therapeutics

**DOI:** 10.3390/ph16121652

**Published:** 2023-11-26

**Authors:** Sandeep Paudel, Neha Mishra, Rajesh Agarwal

**Affiliations:** Department of Pharmaceutical Sciences, Skaggs School of Pharmacy and Pharmaceutical Sciences, University of Colorado Anschutz Medical Campus, Aurora, CO 80045, USA; sandeep.paudel@cuanschutz.edu (S.P.); neha.mishra@cuanschutz.edu (N.M.)

**Keywords:** cancer, phytochemicals, immunomodulation, therapeutics

## Abstract

Phytochemicals are natural plant-derived products that provide significant nutrition, essential biomolecules, and flavor as part of our diet. They have long been known to confer protection against several diseases via their anti-inflammatory, immune-regulatory, anti-microbial, and several other properties. Deciphering the role of phytochemicals in the prevention, inhibition, and treatment of cancer—unrestrained cell proliferation due to the loss of tight regulation on cell growth and replication—has been the focus of recent research. Particularly, the immunomodulatory role of phytochemicals, which is pivotal in unchecked cell proliferation and metastasis, has recently been studied extensively. The immune system is a critical component of the tumor microenvironment, and it plays essential roles in both preventing and promoting oncogenesis. Immunomodulation includes stimulation, amplification, or inactivation of some stage(s) of the immune response. Phytochemicals and their products have demonstrated immune regulation, such as macrophage migration, nitric oxide synthase inhibition, lymphocyte, T-cell, and cytokine stimulation, natural killer cell augmentation, and NFκB, TNF, and apoptosis regulation. There is a dearth of extensive accounts of the immunomodulatory effects of phytochemicals in cancer; thus, we have compiled these effects with mechanistic aspects of dietary phytochemicals in cancer, highlighting promising candidates and ongoing clinical trials on immunotherapeutic strategies to mitigate oncogenesis.

## 1. Introduction

Cancer is a disease of abnormal and unchecked cell growth and proliferation, and it remains the second leading cause of substantial mortality and morbidity rates worldwide [1,2,3,4,5]. According to the American Cancer Society, approximately 609,360 people died due to various form of cancers, with around 1.9 million new cancer cases reported in 2022 [5]. As cancer involves unchecked cell growth, a prominent aspect of cancer prevention and treatment is modulating the immune response. Refurbishing the immune system to defeat and minimize the chances of cancer recurrence is essential in cancer treatment [6,7]. The immune system is the key component responsible for proper cell growth and proliferation while controlling metastasis [8]. Experiments have shown that the immune system can react to experimentally induced tumors in animal models. Thus, it can be concluded that the host cell immunological network can modulate malignant cells [9,10]. Research shows that immunotherapeutic manipulation can control the spread of tumors of almost all types [11].

Local interventions and systemic therapies, like surgery, chemotherapy, radiation, hormonal therapy, and other targeted therapies, are common approaches to augment the immune system for treating cancer. Chemotherapy is a major consideration, and is one of the most widely used treatments in both early and advanced stages of cancer; however, due to its highly cytotoxic nature and other severe side effects, new approaches with minimal iniquitous effects are warranted [12]. A successful shift towards phytochemicals may diminish the side effects and be cost-effective, thus, playing an important role in the socio-economic sustainability of cancer therapeutics as well. Research endeavors are focused on understanding the drawbacks of modern chemotherapies, reevaluating and rediscovering the anticancer role of traditional medicine and phytochemical substitutes (natural products) that are safer to use and are on the rise [13]. Various clinical trials are underway to assess potent phytochemicals with anticancer and immunomodulatory effects for the comprehensive therapy of specific types of cancer [13].

Phytochemicals encompass a broad range of natural compounds obtained from flora or plant products, such as carotenoids, phenolics, alkaloids, nitrogen and sulfur-containing compounds, to name a few [14]. Since ancient times, plant sources have been an integral part of our diet owing to their nutritional and medicinal values. Enormous volumes of evidence show that phytochemicals can diminish the risk of several chronic diseases and conditions involving a large immunological component, including, but not limited to, cancer, diabetes, cardiovascular diseases, and arthritis [15,16,17]. Therefore, the intake of phytochemicals such as curcumin, quercetin, flavonoids, luteolin, apigenin lycopene, epigallocatechin-3-gallate (EGCG), resveratrol, curcuminoid, silibinin, and soybean in the diet may be more useful for cancer prevention. The anticancer effect, along with reduced side effects, immunomodulatory functions, and antioxidant properties of phytochemicals, make them a more considerable preventive measure against cancer initiation and recurrence.

There are several advantages to including phytochemicals as an essential part of the diet. The foremost is the ease of administration, particularly for palatable compounds in their natural form, such as fruits, raw vegetables, nuts, or as part of cuisine, such as spices, oils, herbs, or processed forms such as vines, non-alcoholic beverages, and chocolate. Routine and mindful intake of phytochemicals can have cancer-preventive properties. Additionally, the consumption of whole bio-products has been shown to be more effective than the isolation of one or a few active compounds. For example, bitter guard juice is more effective in preventing pancreatic cancer [18], and whole fermented rice bran is more effective in preventing colon cancer [19]. This review focuses on selected phytochemicals that can be administered orally or consumed as part of the diet, containing anticancer and immunomodulatory properties to fight against a vast variety of cancer and the mechanistic aspects of immunological regulation by these phytochemicals.

## 2. Phytochemicals: History and Classification

Phytochemicals are biologically active natural compounds derived from plants (Greek word, phyto, meaning plants) that provide macro- and micronutrients to humans [20]. In plants, they are responsible for providing aroma, color, taste, and protecting against environmental hazards and pathogenic attack [21]. Phytochemicals may be available in the form of secondary plant metabolites, known for their nutritive and protective role in human health [22]. Phytochemicals have been in existence since the emergence of plants, and their active roles have been known since ancient times. However, knowledge of the chemistry of compounds conferring medicinal properties to phytochemicals was gained a few hundred years ago. The earliest records of herbal medicines goes back to around 2800 BC, written by the Chinese emperor Shen Nung in “The Great Native Herbal,” and it gives an account of cancer treatment through immunomodulation [23]. Ancient Indian literature also shows the use of medicinal plants in the form of Ayurveda, for tumor management via inflammation and immune response management [24]. Later, this medicinal knowledge was brought to Egypt and Europe, first by Hippocrates (460–377 BC) and then by Aristotle (384–322 BC) [25]. The journey of medicinal plants, i.e., utilizing plant product(s) as a source of medicine, began in 28 A.D. by Greek physicians, as indicated in De Materia Medica [26]. Later, salicin, isolated from the same willow tree, was used as an anti-inflammatory and pain-relieving drug [27]. During the 1980s, many laboratories started to identify phytochemicals as medicines. Medicinal plants from traditional sources have been increasingly used for the search of new drugs. After decades of active use as traditional medicine (herbal preparation), the isolation of the first phytochemicals, i.e., alkaloids (quinine, morphine, strychnine), was successful in the early 19th century, which started a new era for research on dietary and medicinal plants [23]. Phytochemicals are known to possess several biological properties such as anti-microbial activity, repair of cells, antioxidant properties, and the inhibition of different cancer growths [28]. Phytochemicals are mainly present in fruits, seeds, roots, stems, leaves, and flowers [29].

There are no strict classification guidelines for phytochemicals. One way to classify them is according to their functional in plant metabolism; phytochemicals are classified as primary and secondary metabolites. Primary metabolites are necessary for plant life and include carbohydrates, proteins, lipids, nucleic acids, and their building blocks. Secondary metabolites are the remaining plant chemicals produced through the metabolism of primary metabolites by cellular activity. The most common classes of phytochemicals according to their chemical structural identity are phenolics (45%), terpenoids and steroids (27%), alkaloids (18%), and other chemicals (10%) [30]. A brief overview of these phytochemical classifications is provided below.

### 2.1. Phenolics

Phenolic phytochemicals are the largest group of phytochemicals present in the plant kingdom [31]. They have a hydroxyl group (-OH) with a covalently bonded aromatic hydrocarbon group, for example, C6H5OH (phenol). They form a diverse group that includes hydroxybenzoic and hydroxycinnamic acids. These are secondary metabolites synthesized by the phenylpropanoid, shikimate, and pentose phosphatase pathways in plants. Polyphenols are present in various parts of plants and play important role in growth, pigmentation, structure, and defense [32]. Within this group, the most important dietary phenolics are flavonoids, phenolic acids, and polyphenolic amides. The structural background, dietary source, and medical plant source of the most studied flavonoids and phenolic acids are listed in Table 1.

### 2.2. Terpenoids

Terpenoids are a major class of secondary metabolites that contain carbon backbones made of isoprene (2-methylbuta-1,3-diene) units [38]. The generic name “terpene” means hydrocarbons found in turpentine, and the suffix “ene” means the presence of an olefinic bond (containing two isoprene units, hence ten carbon atoms) [39]. Terpenoids are produced by a wide variety of plants, animals, and microorganisms, and their roles in living organisms can be grouped as functional, defensive, and communicative [40]. Based on the isoprene units, terpenoids are divided into various groups such as hemiterpenoids, monoterpenoids, sesquiterpenes, diterpenes, triterpenes, and tetraterpenoids [41]. The major dietary and medicinal sources of terpenoids are stated in Table 2. Among the various groups of terpenoids, diterpenoids are mainly used for cancer therapy [42].

### 2.3. Alkaloids

Alkaloids are natural secondary metabolites derived from plants, fungi, and animals (~3000 distinct alkaloids have been characterized) [45]. They are low molecular weight heterocyclic nitrogenous compounds (with one or more nitrogen atoms present as part of a ring of atom called a cyclic system), which are colorless, crystalline, non-volatile and have low toxicity with higher stability [46]. They are further classified according to the amino acids from which they are derived in the biosynthetic pathway. The major classes of alkaloids are pyrrolidine, pyridine-piperidine, quinoline, isoquinoline, and pyrrolidine-pyridine, as stated in Table 3. Alkaloids are mainly used by plants for defense against microorganisms and insects by producing allelopathically active chemicals [47]. Alkaloids have a restraining effect on the topoisomerase enzyme, leading to stalled DNA replication and cell death, and have various pharmacological activities, including anti-cancer properties, apart from anti-bacterial and anti-inflammatory effects [48]. Alkaloids derived from plants have significant efficacy in the suppression of oncogenesis.

## 3. Cancer Microenvironment: Immunological Milieu

The microenvironment of normal healthy cells incudes immune cells, fibroblasts, blood and lymphatic vessels, and interstitial extracellular matrix [59]. This cellular machinery plays a central role in maintaining tissue homeostasis and functions as a barrier to tumorigenesis [60]. Aberrant signaling from messengers such as chemokines, cytokines, reactive oxygen species (ROS), and lipid mediators, indicating a polarized microenvironment and altered tissue homeostasis, may initiate/promote tumorigenesis and growth. However, the actual underlying mechanisms for oncogenesis, particularly owing to immune responses, are not well elucidated for most cancer types. The tumor microenvironment (TME) contains both malignant and nonmalignant cells, where non-malignant cells bear the initial tumor-promoting role. The ‘seed and soil’ hypothesis provides significant insights into the relationship of the TME and malignant tumor, where TME is the ‘soil’ and is crucial for the tumor or ‘seed’ to germinate and further grow [61]. Tumors do not resemble malignant cells of only one type but are complex organoids where various cells are recruited and transformed [62]. TME, along with malignant cells, contains various immune cells, tumor vasculature, fibroblasts, lymphatics, pericytes, and adipocytes [63]. The TME initially primes the immune system by the infiltration of immune cells that send chemical signals masking tumor antigens, thus protecting the cancerous cells [64]. At the cancer site, stromal cells release various mediators and cytokines to participate in immune regulation.

### 3.1. Overview of the Immune System in Cancer

The immune system is an association of complex networks of specialized molecules, cells, tissues, and organs that provides defense from foreign pathogens, aberrant cells, and tumors. The main physiological function of the immune system is to distinguish between “self”, “non-self,” and “altered-self” structures or transformed cells [65]. Cells of the immune system are derived from hematopoietic stem cells by hematopoiesis, belonging to either the lymphoid lineage (B and T cells, natural killer [NK] cells, and innate lymphoid cells) or the myeloid lineage (granulocytes, basophils, eosinophils, neutrophils, monocytes-macrophages, and dendritic cells [DCs]) [65]. Molecules that can be recognized by the immune system are considered antigens, and are generally presented on the surface of target cells [66]. Based on antigen specificity, effector responses, and kinetics of activation, the immune system is divided into two distinct components: innate and adaptive immunity [67]. The immune system acquires the ability to recognize, detect, and eliminate different tumors, even though the human body is not completely resistant to cancer [68]. Moreover, tumor cells are regulated by a dynamic process called immunoediting [69]. During this process, innate and adaptive immune cells are triggered by inflammation in the tumor, recruiting immune cells to the arising tumor and synthesizing cytokines and chemokines. The functions of innate and adaptive immune responses in cancer are important for understanding the effect of phytochemicals on cancer and are detailed below.

#### 3.1.1. Innate Immune Response in Cancer

The innate immune system, also known as the natural immune system, recognizes foreign pathogens or non-self-structures based on receptors encoded in the germline known as pattern recognition receptors [70,71]. Innate immunity is the first line of defense in the body [72]. It plays a critical role in cancer, as innate immune cells can directly interact with tumor cells for elimination [73]. NK cells are principally responsible for killing MHC-lacking cancer cells. Upon activation of stimulatory receptors, NK cells express inflammatory cytokines such as interferon gamma (IFN-γ) and perforins, activating the apoptotic pathway in tumor cells [74]. IFN-γ can also interact with other receptors on tumor cells via Fas ligand, tumor necrosis factor (TNF), TNF-related apoptosis-inducing ligand (TRAIL), and lymphotoxin alpha, which in turn enables apoptosis [75,76]. NK cells have two subtypes: one population expressing CD56dim, CD16bright, and the other CD56bright, CD16dim surface proteins [77]. The population with high CD16 expression shows cytotoxic properties, whereas low expression shows immunoregulatory properties for killing tumor cells without immunization [78]. Apart from these receptors, NK cell activity is modulated by several cytokines, such as IL-2, IL-12, IL-15, IL-18, and IL-2 [65,67,79,80,81,82]. Apart from this, innate cells such as NK cells, DCs, and lymphoid cells have an important function of presenting antigens to T cells through major histocompatibility (MHC), connecting the link between the innate and adaptive immune systems [83] and discovering trained immunity.

**Figure 1:**
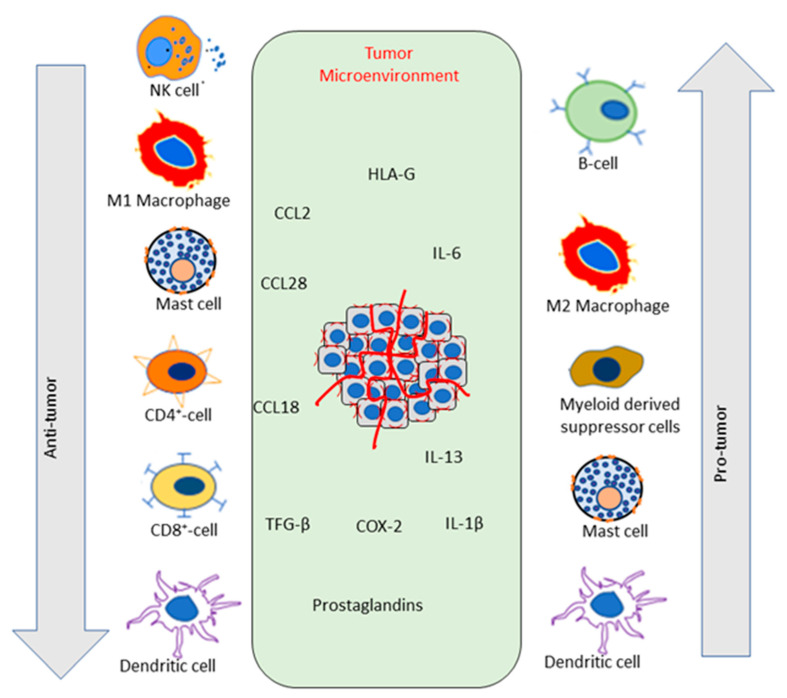
Recruitment of different immune cells to the tumor site and their anti-tumor and pro-tumor properties. Red lines indicate blood vessels.

Tumor cells enhance the expression of chemical messengers such as chemokine C-C motif ligand (CCL)-2, CCL28, CCL18, TGFβ, cycloxygenase-2, prostaglandins, IL-1β, IL-6, IL-13, and human leukocyte antigen G [84]. These secretary proteins recruit immune suppressor cells, TregS, tumor-associated macrophages (TAMs), myeloid-derived suppressor cells (MDSCs), mast cells, NK and NKT cells, tumor-associated DCs that aggregate around the tumor and inhibit immune surveillance, as shown in Figure 1. MDSCs are intermediate cell types between myeloid progenitors and terminally differentiate cells, functionally known for their suppressive activity towards T cells [85]. However, there persist some reports that MDSCs may not be a distinct class or intermediate subtype, with functions overlapping with neutrophils. The role of MDSCs, their functional and antigen presentation properties, as well as their functions in the TME, are reviewed in detail by Engblom et al. [86].

**Figure 2:**
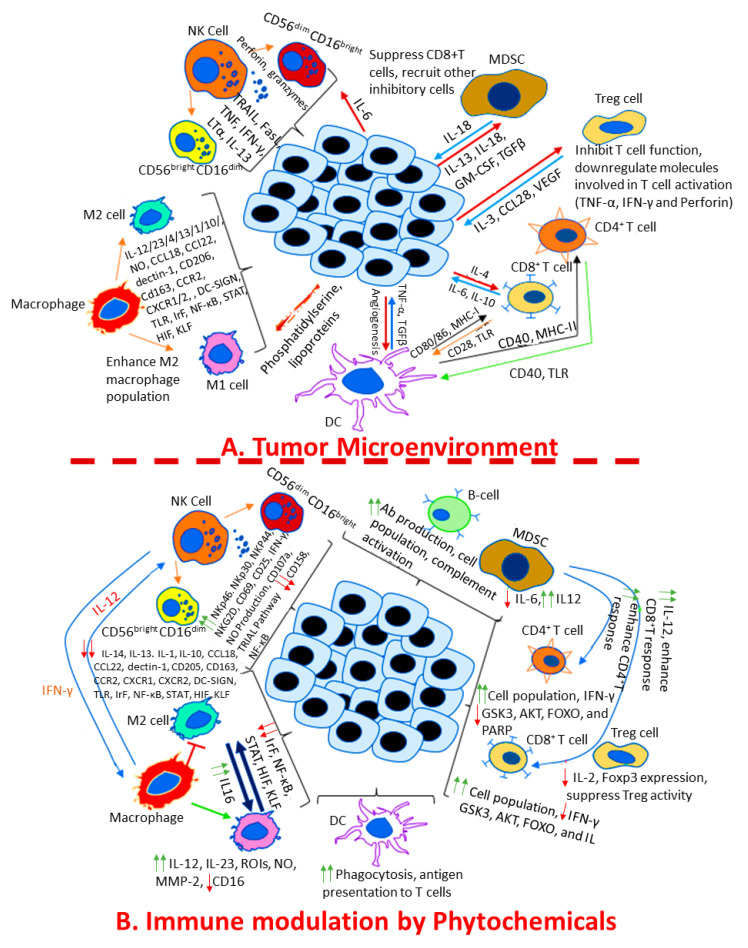
Overview of immune-modulation of the cancer microenvironment by phytochemicals. The green arrows represent upregulation and red arrows represent downregulation.

The pleiomorphic nature of cytokines in the TME contributes to promoting cancer cell proliferation, bypassing apoptosis, inducing EMT of cancer cells, and facilitates tumor tolerance, angiogenesis, invasion, and metastasis [87]. Angiogenic processes depend on the tight coordination and balance between positive and negative modulators through the action of various molecules, enzymes, cellular junction proteins, and various adhesion receptors [88]. Tumor angiogenesis downregulates as well as shift the balance from negative to positive regulators [89]. This process involves sequential effects that primarily include endothelial cell sprouting, loss of mural cell-endothelial cell association, increased vascular permeability and density [90]. Recent studies have implicated tumor-infiltrating immune cells as crucial mediators of cancer initiation and progression [91]. The inflammatory response triggered by immune cells finally leads to enhanced endothelial cell activation, proliferation, and vascular burgeon [92]. These immune cells comprise cells of both innate and adaptive immunity. The innate cells consist of macrophages, granulocytes, mast cells, NK cells, and DCs. Within these cells, mast cells and macrophages recruit additional leucocytes by secreting soluble cytokines and chemokines and recruit T cells and B cells resulting in an immune response, as stated in Figure 2A, which finally contributes to tumor progression and affect its therapy.

Macrophages are another cell type of innate immunity that counter tumor cells. Tumor cells are known to express surface molecules like phosphatidylserine and low-density lipoproteins, which boost the activation of macrophage-induced phagocytosis [93]. Within the TME, TAMs influence tumor progression, extracellular matrix remodeling, proliferation, invasion, and angiogenesis. Macrophages are mainly of two types: M1 and M2, which play a role in the polarization of Th1 and Th2 T cells, respectively [94]. Activation of M1 enhances the production of IL-12, IL-23, ROS, and NO [95]. M2, on the other hand, enhance stimulation of IL-4, IL-13, IL-1, IL-10, CCL18 and CCL22, dectin-1, CD206, toll-like receptor (TLR)-1, TLR-6, and TLR-7 [96], scavenger receptor A, scavenger receptor B-1, CD163, CCR2, CXCR1,CXCR2 and DC-specific intercellular adhesion molecule-3-grabbing non-integrin, as shown in Figure 1 [97,98,99]. Different factors influence the M1 to M2 polarization, such as interferon regulatory factor (IrF), NFκB, STATs, hypoxia inducible factor (HIF), and Kruppel-like transcription factor [100]. For example, in the case of melanoma cells, melanomas exosomes produce HIF-1α and HIF-2α in M1 and M2 macrophages, respectively. Higher counts of TAMs are detected in different types of tumors. In malignant mammary tumors, CD206 expressing M2 macrophage infiltration is higher, whereas in benign tumors, infiltration of M1 is found higher, but within TME, there is a phenotyping shift for macrophages from M1 to M2, leading to cancer progression [101]. Various reports have indicated that pro-inflammatory cytokines released by M1-macrophages inhibit the proliferation of tumors, whereas M2-associated cytokines are involved in tumor growth [102,103]. Wang Y et al. showed that IL-12 within TME can promote macrophage from M2 to M1 to overcome tumors [104]. The inflammatory cytokine IL-6 is critical to polarize M2 through the mTOR signaling complex 2 (mTORC2) and Akt, promoting tumor growth and metastasis [105]. Various research findings highly suggest M2-macrophages’ negative role in TME; thus, M2 polarization inhibition can stop tumor progression [106].

DCs can interact with tumor cells through integrins and other receptors, finally leading to the phagocytosis of apoptotic cancer cells. Furthermore, DCs are professional antigen-presenting cells, which play crucial role in interlinking innate and adaptive immunity [107]. DCs are abundantly present in TME in various cancers such as lungs, breast, head and neck, colorectal, renal bladder, ovarian, and gastric [108]. Tumor burden is directly linked with the number of DCs in various cancers; for example, in ovarian cancer, as the tumor progresses, the number of tumors infiltrating DCs increases [109]. Within TME, DCs switch from immune stimulatory to immune suppressive DCs with the upregulation of immune suppressive molecules and decrease of T cell infiltration [109]. Sisirak et al. showed that in breast cancer, DCs are associated with worse prognosis. They show a poor response to TLR stimulation in respect to antigen presentation, as well as low IFN production and sustained FOXP3+ Treg expansion [110].

#### 3.1.2. Adaptive Immune Response in Cancer

In comparison to the innate immune presenting system, the adaptive immune system is a slower immune response but is more specific in nature [111]. Thus, adaptive immunity is an important power line of defense with immunological memory and high specificity. The effector functions of the adaptive immune system are mediated by the expression of specialized receptors such as B cell receptor and T cell receptor. During the process of development, these receptors undergo somatic recombination; as a result, diverse antigens have the capacity to bind to these receptors [112]. The important hallmark of B and T cells is, upon antigen recognition, they can undergo the process of clonal selection, which facilitates the eradication of threats [65]. The key cells in this group are T-lymphocytes and B-lymphocytes [113]. B-lymphocytes are antigen-presenting cells, which have the ability to neutralize, agglutinate foreign cells, precipitate serum antigens, and activate the complement to produce antibodies [114]. The T-lymphocytes, on the other hand, can produce several types of cytokines, which are important activators of other immune cells. Adaptive immune cells mainly interact with tumor cells via tumor antigens by antigen presentation to eliminate tumors [115]. The TME is densely packed with infiltrating CD8+ cytotoxic T cells, CD4+ helper T cells, and FOXP3+ regulatory lymphocytes in various cancers such as bladder [116], renal [117], ovarian [118], prostate [119], skin, and various solid tumors [120]. Activation of T cells is regulated by specific MHC molecules in coordination with ligands of their costimulatory molecules like CD40, ICOS, GITR, OX40, and 4-IBB [121]. In addition, various cytokines such as IL-2, IL-10, IL-15, IL-17, and TGFβ play a role in T-cell function for its antitumor response [122,123]. Within TEM, CD4+ helper T cells are critical cells that are important in the recognition of neoantigens and interaction with DCs through CD40L enhancing CD8+ T cell priming and activation [124,125]. CD4+ T cells, in the presence of TGFβ and IL-10, can differentiate into inducible Treg cells, which are a subset of CD4+ T cells; within different tumors, they suppress antitumor properties of CD4+ and CD8+ T cells leading to poor prognosis and increased tumor growth (152). Research has shown that Tregs infiltrating tumors also have inhibitory molecule expression, such as CTLA-4, PD-1, and LAG-3, compared to peritumoral Tregs [121]. Cytokines IL-12 and IL-6, on the other hand, can inhibit the effector function of CTLs against tumors as well as stimulate the role of Tregs [126]. In addition, Tregs inhibit the function of most immune cells present in TEM like macrophages (promote M2 phenotype), NK cells, DCs, B cells, and CD4+ and CD8+ T cells and produce immune suppressant molecules like IL-1, ROS, VEGF, and TGFβ [127,128,129]. In respect to other subsets, CD8+ T cells infiltrating TME have shown to reduce tumor progression with their enhanced ability to produce IFN-γ (pro-inflammatory cytokine) [130,131]. IFN-γ shows an array of functions such as differentiation of T cells to Th1 cells, differentiation of CTLs to effector CTLs, inhibition of angiogenesis, promotion of adaptive immunity, and induction of anti-metastatic activity of IL-12 [132,133,134]. Tumor cells, on the other hand, can regulate T cell function where it decreases the IFN-γ release, producing immune escape mediators like STAT3, PD-L1, and IDO1 [134,135,136].

## 4. Role of Phytochemicals in Modulating Immune Functions in Cancer

Phytochemicals have remarkable anti-cancer properties that have been demonstrated at both in vitro and in vivo levels. Phytochemicals confer protection from malignancy through scavenging free radicals, reducing invasion and angiogenesis, and suppressing proliferation of tumor cells [137]. In addition, they show their activity on different molecular targets, membrane receptors, kinases, tumor activator proteins, transcriptional factors, cyclins, caspases, microRNAs, and signal transduction pathways [91].

### 4.1. Regulation of the Innate Immune Response in Cancer by Phytochemicals

Phytochemicals have a great ability to modulate the immune response by regulating immune cells. Several phytochemicals, alone or in combination, are crucial in the stimulation, activation, and maintenance of T cell and NK cell cytotoxicity. Silibinin has been shown to increase the number of CD4+ and CD8+ T cells and neutrophils but decrease macrophage and MDSCs cell numbers in 4T1 luciferase-transfected mammary cancer in female BALB/c and CB17-Prkdc Scid/J mice [138]. Quercetin-triggered NK cell-mediated tumor cell apoptosis through the NKG2D-activating receptor in quercetin-treated K562, SNU1, and SCN-C4 cells, also affected the Th1/Th2 ratio in tumors [139]. Apigenin increases CD4+, CD8+ T cell numbers and reduces suppressive Treg cell numbers in mice [140].

Fraker et al. showed that the oral administration of retinol in wild-type BALB/c and congenitally athymic BALB/c mice can enhance the cytotoxic activity of NK cells in the spleen within an hour of treatment [141]. The enhanced activity is associated with increased expression of the retinoic acid early-inducible gene, and its products act as ligands for the NK cell surface receptor NKG2D [142]. Phytochemicals are not only involved in cytotoxic activity but also play a role in NK cell maturation and increase the expression of activating receptors of NKp46, NKp30, NKp44, NKG2D CD69, and CD25 and IFN-γ and downregulate the inhibitory receptor CD158, in both in vitro and in vivo mouse studies [143,144]. Isoflavone (genistein), even at low concentrations (0.5–1.0 μmol/L), enhances NK cell degranulation and its activity in vitro [145,146].

Curcumin shows immunomodulatory effect upon nitric oxide (NO) production by NK cells as well as macrophages causing cytotoxicity against tumor cells like in AK-5, YAC-1, and breast tumor exosomes [147,148]. Fiala et al. showed that curcumin, in combination with omega-3 fatty acid, enhances NK cell-mediated apoptosis by inhibiting NFκB signaling in pancreatic cancer both in vivo and in vitro [149]. In-depth immunological studies revealed that generally tumor cells decrease the CD4+/CD8+ ratio and inhibit T-cell functions to escape from immune surveillance [8]. Curcumin was also shown to inhibit immunosuppressive Treg cell functions, downregulate IL-10 and TGFβ secretion, and modulate the macrophage and DC cell functions in both in vitro and in vivo models [150].

Phenolic compounds, like resveratrol, showed increased toxic effects against various cancer cell lines such as leukemia (K562), human promyeloblastic leukemia (KG-1a), HepG2, and A549 [151]. The mechanism of toxicity was correlated with an increase in phosphorylation of JNK, ERK-1/2, and RK1/2 MAP kinase activity, perforin, NKG2D, and IFN-γ upregulation, TRAIL pathway activation, CD107a expression, CD8+- and CD4+-T-cells stimulation, and inhibition of constitutively active signal transducers and activators of STAT3 signaling [152,153,154]. The TRAIL pathway has been shown to mediate apoptotic cell death in various cancers, such as human prostate carcinoma, breast, colon, skin, and neuroblastoma both in vitro and in vivo studies, as shown in Table 4 [155,156,157,158,159].

Phytochemicals also regulate macrophages to achieve anti-tumor activity, not only by inhibiting HIF-1α and HIF-2α but also by maintaining a proper balance in M1 and M2 polarization [177]. The polyphenolic compound resveratrol, in humans (PBMCs), has been shown to trigger pro-inflammatory signaling in macrophages [178]. In M1 cells, it stops the increase of pro-inflammatory molecules by downregulation of CD16 and upregulation of metalloproteinase (MMP)-2, whereas in M2 cells, it stops the increase of proangiogenic molecules by upregulation of CD14, MMP-2, and MMP-9 and downregulation of endocytosis, as shown in Figure 1 [178]. Fenretinide, a derivative of retinoic acid, has been shown to inhibit M2 macrophage polarization in colon cancer in APC ^min/+^ transgenic mice, with an adverse impact on cancer attenuation [179]. IL-4, IL-13, CD206, Fizz1, and PPARγ protein levels are blocked by fenretinide, which finally inhibits M2 polarization without any effect on M1 polarization. Cannabinoids can modulate macrophage activity by reducing the expression of TNFα and IL-1β, suggesting its anticancer potential in xenograft tumors for colorectal cancer cells (HTC116, SW480, SW620, and HT29) [162,180]. Echinacea, on the other hand, enhances cytokine production (TNF-α, IL-1, IFN-β), activates macrophages by increasing the expression of CD80, CD86, MHCII molecules, and phagocytosis in murine bone marrow-derived macrophages [165,181].

Other crucial innate cells interacting with the tumor cell are DCs. Curcumin treatment in male C57BL/6 mice strongly downregulates CD80, CD86, and MHC class II expression, but not MHC class I expression on DCs. The DCs also exhibit impaired IL-12 expression and pro-inflammatory cytokine production (IL-1β, IL-6, and TNFα). Apigenin treatment leads to low expression of PD-L1 in DCs, resulting in enhanced T cell immunity in the melanoma xenograft mouse model and human peripheral blood mononuclear cells [182].

### 4.2. Regulation of Adaptive Immunity in Cancer by Phytochemicals

Phytochemicals have a strong effect on B cell and T cell populations and play integral regulatory roles in maintaining and enhancing the adaptive immune response. In vivo studies with mice inoculated with Ehrlich’s ascites mammary carcinoma (EAC) have shown that phytochemicals such as curcumin, even at low doses, increase T cell population, decrease tumor growth, increase cytotoxic activity of CD8+ T cells with an increase in IFN-γ release, and increase in CD4+ T cell and B cell populations [183,184]. Phytochemicals have also been shown to inhibit tumor cell proliferation by activating the apoptosis pathway and caspase-3 activity via inactivation of PI3 kinase targets such as GSK3, AKT, FOXO, and PARP degradation [185], as shown in Figure 2. Shao et al. showed that bisdemethoxycurcumin can suppress bladder cancer by enhancing CD8+ T cell infiltration in the TME and increasing the level of IFN-γ by reducing the MDSC population in salivary gland tumor cells in BALB-neuT mice [186,187]. They have also shown that MDSCs, on one hand, reduce IL-6 while on the other hand, induce IL-12, enhancing the CD4+ T and CD8+ T response. Additionally, curcumin inhibits the Treg suppressive activity by inhibiting IL-2 secretion and decreasing Foxp3 expression [188].

Rocaglamides act as immunosuppressive phytochemicals by inhibiting the production of IL-2, IL-4, and IFN-γ in T cells circulating in peripheral blood [189]. Garlic extract, which contains various phytochemicals such as allicin, alliin, diallyl disulfate, diallyl trisulfide, ajoene, and s-allyl cystine, was shown to increase the ratio of CD4+/CD8+, enhance the production of IFN-γ in splenocyte of fibroblast tumors, and increase IFN- γ, IL-2, IL-4 levels in breast cancer in Wistar rats [161]. This overall increase in Th1 and Th2 response promoted lymphocyte proliferation [161]. Kis et al. have shown that cannabinoids have a protective role in cancers of various regions, including breast, lung, colon, prostate, skin, and brain, by enhancing the effect of T cells and decreasing the production of T-helper 2 cytokines such as IL-10 in female athymic nude mice xenograft tumors with MCF-7, MDA-MB-231, DU-145, CaCo-2, and AGS, as shown in Table 4 [162]. Tinospora cordifolia greatly affects the proliferation of B-lymphocytes and T-lymphocytes subsets (CD4+ and CD8+) and the secretion of Th1 and Th2 cytokines in colon, cervical, and oral squamous carcinomas [170,171]. Apigenin, in the case of melanoma (melanoma xenograft model with A375, A2058, and RPMI-7951), strongly suppresses the IFN-γ-induced activation of STAT1, leading to decreased PD-L1 expression; thus, sensitizing them to T cell-mediated killings [172]. Studies have shown that apigenin potentially stabilized Ikaros expression by targeting CK2 [173]. Overall, various phytochemicals can differentially modulate various functions of innate and adaptive immune cells to overcome tumor growth, as shown in Table 4.

### 4.3. Phytochemicals in Cancer: Clinical Trials and Other Studies with Human Patients

Effective clinical trials of different phytochemicals, such as various alkaloids and terpenes, flavopiridol, curcumin, silibinin, and resveratrol for the treatment of different disease conditions, are ongoing [26,190,191,192,193,194]. Various phytochemicals, not only in pre-clinical studies but also in several phases of clinical trials, have shown major positive outcomes. For example, in phase I clinical trials, curcumin alone (8 g/day) or in combination with quercetin (400/20 mg) showed significant measurable histological improvement in patients with various cancer such as pancreatic cancer [195], oral leukoplakia [196], cervical intraepithelial neoplasia [195], multiple myeloma, and advanced colorectal cancer [196]. In a phase II clinical study, Carroll and colleagues showed that curcumin (4 gm/day) resulted in a 40% reduction in aberrant crypt foci within 30 days of treatment [197].

Resveratrol clinical trials, with a dose of 2.5 gm/day for the period of 29 days, reduced the concentration of insulin-like growth factor (IGF)-1 and IGF-binding protein 3 in the plasma, inhibited tumor formation, and metastasis [198,199]. Resveratrol with dose of 1 gm/day for four weeks decreased cytochrome P450 and CYP3A4 levels and increased CYP1A2 in the plasma of healthy individuals, which could aid in cancer prevention. These molecules play an important role in detoxification and carcinogen inactivation [200]. Resveratrol shows its therapeutic activity by promoting NK cells’ effector function via recognizing transformed cells prior to proliferation more rapidly than T cells. NKG2D, an antigen receptor expressed on NK cells, T cells, and CD8^+^ cells, recognize specific ligands expressed on transformed cells for their tumor suppressive effect [201,202].

In addition to curcumin and resveratrol, catechins such as EGCG and epicatechin-3-gallate (ECG), present in green tea in high concentrations, prevent DNA damage and mutagenesis in healthy cells [203,204]. Liver cancer patients or chain-smokers have elevated levels of 8-OHdG in their urine. Green tea supplement of 500–1000 mg/day for a period of three months significantly decreases 8-OHdG levels [204,205,206]. Zheng et al. showed that green tea had positive outcome with prostate cancer patients as well; however, green tea did not have a significant outcome in patients with stomach cancer [207,208]. Clinical trials studying elderly men supplemented with β-carotene increased CD3+, CD4+, CD8+ T cell percentages and enhances NK cell numbers, and EGCG with DNA vaccine enhances CD8+ cell-mediated immune responses in the TME [209]. In contrast, β-carotene (15 mg/day) and retinol (25,000 IU/day) enhance lung cancer and its mortality [210]. Another study showed that the intake of β-carotene (15 mg/day), α-tocopherol (30 mg/day), and selenium (50 µg/day), for a period of five years, lowered the risk of gastric cancer, although no effects were observed with esophageal cancer [210,211]. These conflicting outcomes of the same phytochemicals may be due to certain biomarkers expressed in different cancers during their progression. It was demonstrated that treatment with phytochemicals regresses tumor volume and enhances the synergistic effect of various chemotherapeutic drugs like 5FU, doxorubicin.

Ishikawa et al. [212] showed that in patients with colorectal, liver, or pancreatic cancer, daily consumption of 4 aged garlic capsules/day (Allium sativum 500 mg) for 12 weeks led to increased NK cell numbers and activities and was associated with more favorable outcomes. Further, daily consumption of 6 capsules (2.5 mL garlic) for 1 year led to decreased colon adenoma size and numbers [213]. A list of all the clinical trials and other studies with human subjects on the efficacy of phytochemicals in treating cancer is provided in Table 5.

## 5. Challenges and Future Prospectives

Although phytochemicals have been extensively studied in various cancer cell lines in vitro and in pre-clinical animal models, as well as clinical trials with some promising natural therapeutics ongoing, their clinical efficacy as an anti-cancer target is still under debate. The major problem associated with phytochemical drugs is their solubility and adsorption, beginning in the oral cavity and continuing up to the gut milieu. Additionally, phytochemicals can have a wide range of pharmacokinetics that hinge on a multitude of factors, such as the type of compound and mode of preparation. Therefore, the synthesis of easily soluble phytochemical analogs, with well-characterized kinetics, mainly in aqueous or other stabilizing conditions, that can be absorbed effectively in the gut is warranted. Furthermore, the half-life of phytochemicals is very short in human blood, and the mechanisms of their metabolism remain unclear. Therefore, improving the bioavailability and stability of phytochemicals in vivo represents another challenge to researchers. In this consequence, more efficient research is required to increase the longevity of their half-life and thereby efficacy, and a better and more in-depth understanding of their mechanism of metabolism and subsequent activity is needed.

Unfortunately, no phytochemical has been established or approved to combat cancer. Challenges associated with phytochemicals, such as inadequate information about specific targets, pre-clinical study data, optimal dose, solubility, and longevity or bioavailability, need to be overcome before phytochemical therapeutics can be approved. There is also a threat of overdosing or toxicity due to the consumption of phytochemicals in very high doses, such as overuse of wine may lead to liver damage, alcohol toxicity, socio-economic problems, and other challenges associated with intoxication. Thus, it is important to consume phytochemicals in limited doses and responsibly, even though most phytochemicals are not known to be harmful in small doses, per the direction of the physician. Additionally, further studies with combination/cocktail phytochemical therapeutics, and cutting-edge basic research with more robust and effective drug development are required. Further understanding of various dietary phytochemicals is necessary regarding various cancers via immune modulation of various innate and adaptive cells within TME, which could be safe, non-toxic, and economical anti-cancer therapeutics.

## 6. Conclusions

Phytochemicals have been reported to play vital immunomodulatory roles in cancer since ancient times. In recent years, extensive research on phytochemicals has shown them to be attractive anti-cancer therapeutics through modulation of the TME. More research is needed for the development of phytochemicals-mediated cancer treatment, through better understanding of cell cycle progression, inhibition of signaling cascades at various stage of cancer progression like initiation, progression, and development.

## Figures and Tables

**Table 1:** Bioactive phenolics, their structural backbones, and sources.

Phenolics	Structural Backbone	Representative Flavonoid	Dietary Sources	Medical Plants	Properties	Refs.
Flavanonol	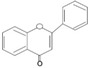	Taxifolin	Tea	*Brysonima crassa*, *Pongamia pinnata*	Antioxidant, anti-inflammatory	[27]
Flavone	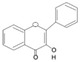	Apigenin, Rutin, Luteolin, Leteolin Glucosides, Chrysin, Apigenin,	Buckwheat, redpepper, fruits and tomato skin, beets, artichokes, lemongrass, chamomile	*Aloe vera*, *Acalypha indica*, *Bocopa moneirra*, *Glyccheriza glabra*, *Limnophila indica*, *Mentha longifolia*, *Momordica charantia*,	Antioxidant	[27]
Flavanols	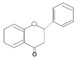	Kaempferol, Quercetin, Tamarixetin, Myricetin, Galangin	Grapefruit, berries, olive oil, red and yellow onion, brassicatees, walnuts	*Azadirachta indica*, *Betula pendula*, *Bauhinia monandra*, *Cannabis sativa*, *Clitoria ternatea*, *Mimosa pudica*	Antioxidant, cardioprotection, antibacterial, antiviral, anticancer	[33,34,35,36,37]
Flavanone	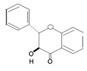	Naringin, Naringenin, Hesperetin, Silybin	Orange, lemon, grapefruit, milk thistel	Citrus media	Antioxidant, antiinflammatory	[31]
Isoflavone	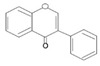	Daidzin, Genistin, Glycitein	Soybean, chickpeas, peanuts, alfalfa sprouts, red clover, soy	*Butea monospermea*	Immunomodulatory, antioxidant	[35]
Flavan-3-ols	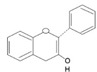	Catechin, Epictechin, Gallate, Proanthocyanidins, Theaflavins, Thearubigins, Epigallocatechin	Black tea, green tea, lentils, wine, cocoas, apple juice	*Atunu raacemosa*, *Camellia sinensis*	Antioxidant, anti-inflammatory, anticancer, immunemodulatory	[31,32]
Hydroxybenzoic acids	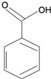	Salicylic Acid, Salicin	Tea, potato, rosaceous fruit, red wine	*Piper marginatum*, *Pandanus Odorus*	Antioxidant	[31]
Hydroxycinnamic acid	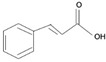	Caffeic, Ferulic Acid, Coumaric Acid	Coffee, apple, plums, cherries, peaches, eggplant, artichoke, cabbage	*Pinuseldarica*, *Rheumemodi*, *cyperus rotundus*, *Euphorbia tirucalli*	Antioxidant, anti-tumor, anti-inflammatory, antimicrobial, antidiabetic	[31,32]

**Table 2:** Bioactive terpenoids, their structural backbones, and sources.

Trepenoids	Structural Backbone	Trepenoids	Dietary Sources	Medical Plants	Properties	Refs.
Hemiterpenoids	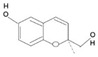	Isovaleric Acid, Prenol, Isoperene	Grapefruit, hops, orange	*Prinsepia utilis*, *Cananga odorata*, *Humulus lupulus*	Antioxidants	[43,44]
Monoterpenoids	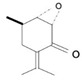	Geranyl Pyrophosphate, Eucalytol, Limonene, Citral, Camphor, Pinene	Mints, garlic, maize, rosemary, ginger, citrus oils	*MenthaLongifolia*, *Anetheumgraveolens*, *Magnolia officinalis*, *Cannabis saativa*, *Cannabis indica*	Antioxidant, anticancer, antidiabetic, immunostimulant	[43,44]
Sesquiterpenes	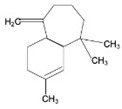	Artemisinin, Bisabolol, Fernesol, Eudesmol	Ceylon cinnamon, pepper, turmeric, ginger, lettuce, and potatos	*Cyperus edulis*, *Aframomumarundinaceum*, *Artemisia annua*, *Thapsia garganica*	Antitumor/anticancer, anti-inflammatory, analgesic, antiulcer, antibacterial, antifungal, antiviral, antiparasitic	[43,44]
Diterpenes	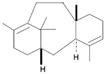	Cembrene, Kahweol, Taxadiene, Cafestol	Coffee	*Coffea arabica*, *Taxusbrevifolia*,	Anti-inflammatory, immunomodulatory	[43,44]
Triterpenes	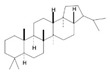	Lanosterol, Squalene, Saponins, Oleanolic Acid, Ursolic Acid, Betulinic Acid	Soyabeans, legumes, alfalfa, java apple, garlic, lavender, caranberries, winged beans, white birch	*Triphyophyllum peltatum*, *Diospyros leucomelas*, *Tetracera boiviniana*	Anticancer, anti-inflammatory, antioxidant, anti-viral, antibacterial, antifungal	[43,44]
Tetraterpenoids	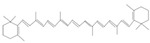	Lycopene, Carotene, Phytofluene, Phytoene	Carrots, pumpkins, orange, sweet potato, orange, autumn olive	*Mauritia Vinifera*, *Myrciaria dubia*, *Spondias lutea*	Anti-inflammatory, anti-ulcer, antibacterial, antiviral, hepatoprotective, immunomodulatory, anti-atherosclerotic, wound healing	[43,44]

**Table 3:** Bioactive alkaloids, their structural backbones, and sources.

Alkaloids	Structure Backbone	Alkaloids	Dietary Sources	Medical Plants	Properties	Refs.
Pyrrolidine	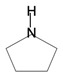	Piperine, Coniine, Isope-lletierine, Preussin B	Barley, bine, peppers, apple, spinach celery, celeriac	*Apium graveolens*, *Spinacia oleracea*, *Malus domestica*, *Capsicum annuum*, *Humulus lupulus*, *Hordeum vulgare*, *Simplicillium lanosoniveum*	Antimicrobial, antitumor, anticonvulsant, anti-tubercular, analgesic	[49]
Pyridine-piperidine	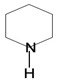	Anabasine	Tobacco	*Anabasis aphyllan*	Antitumor, antimicrobial, antiviral, analgesic, anticonvulsant, antiinflammatory, antioxidant, anti-Alzheimer’s, anti-ulcer, anti-diabetic	[50]
Quinoline	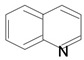	Quinine, Quinidine, Cinchonine, Cinchonidine, Ellipticine	Cocoa, black tea, scotch whiskey	*Cinchona succirubra*, *Ochrosia Elliptica*	Antimalarial, antibacterial, antifungal, anthelmintic, cardiotonic, anticonvulsant, anti-inflammatory, analgesic	[51,52]
Isoquinoline	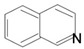	Berberine, Morphine, Montanine, Salsoline, Galantamine	Goldthread, Oregon grape, phellodendron, turmeric, barberry	*Hydrastis Canadensis*, *Papaver somniferun*, *Narcissus tazetta*, *Salsola oppositefolia*, *Hippeastrum Bittatum*	Anti-inflammatory, improves digestion	[53,54,55,56,57]
Pyrrolidine-pyridine	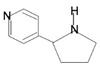	Myosmine, Nicotine	Kiwi, millet, potato, milk, maize, rice, pineapple	*Nicotianatabaccum*	Antitumor, antimicrobial, anticonvulsant, anti-tubercular, analgesic	[58]

**Table 4:** Phytochemicals and their immunomodulatory effects in cancer.

Phytochemicals	Immunomodulatory Effects	Type of Cancer	Study Type	Refs.
Kaempferol	↑↑↑NFκB signaling ↑↑↑IL-1β ↑↑↑TNF ↓↓↓IL6	Skin, liver, colon, ovary, pancreas, stomach, and bladder cancers	In vitro (PBMC and cell HaCaT, THP1-Blue, THP1-Blue-CD14)	[160]
Crude Garlic Extract	↑↑↑CD4^+^/CD8^+^ ratio ↑↑↑IFN-γ ↑↑↑IL-2, IL-4 ↑↑↑Th1/Th2 response ↑↑↑Lymphocyte proliferation	Liver, colon, prostate, and breast cancers	In vivo (Wister rats and chickens)	[161]
Cannabinoids	↑↑↑T-cells and Macrophage ↓↓↓T-helper 2 cells ↓↓↓IL-10 ↓↓↓TNFα and IL-1β expression in macrophages	Breast, lung, colon, prostate, skin, and brain cancers	In vivo (female athymic nude mice) In vitro (cell lines MCF-7, MDA-MB-231, DU-145, CaCo-2, AGS)	[162]
Flaxseed Lignans	↑↑↑NFκB signaling ↓↓↓Proinflammatory cytokines (IL-1ß, IL-6, TNFα, HMGB1, TGFß1, TNFαR1, TGFßR1) ↓↓↓COX-2 level and activity	Breast and prostate cancers	In vivo (female athymic nude mice) In vitro (cell lines MCF-7, MDA-MB-231, DU-145, CaCo-2, AGS)	[163]
Anthocyanin	↑↑↑T-cell proliferation, survival, MDSC differentiation ↓↓↓Cytokine-induced STAT protein phosphorylation	Oral and cervical cancers	PBMCs (healthy adult donors)	[164]
Quercetin	↓↓↓Pro-inflammatory cytokines/chemokines ↓↓↓MHC class II and co-stimulatory molecule ↓↓↓Ag-specific T-cell activation by reducing LPS-stimulated DC activity -Leukocyte biology and Th1/Th2 balance regulation	Oral, cervical, and lung cancers	PBMCs (healthy adult donors)	[6,164]
Echinacea	↑↑↑Macrophages ↑↑↑Phagocytosis ↑↑↑TNF-α, IL-1, IFN-β ↑↑↑Leukocyte mobility ↑↑↑NK cell stimulants and NK cell activation ↑↑↑Murine bone-marrow derived macrophage by increasing CD80, CD86, MHCII expression	Leukemias and lymphomas	In vivo (Leukemic mice)	[165,166]
Curcumin	↑↑↑Apoptosis of malignant cells ↑↑↑T cells ability to kill cancer cells ↑↑↑ CD4^+^ T-cell and B cell numbers ↑↑↑Lymphocyte-mediated immune functions ↑↑↑Progenitor, effecter, and circulating T-cells ↓↓↓Treg cell activity ↓↓↓TGFβ and IL-10 -Th1/Tc1-type cytokine-producing effector T–cell population normalizes in tumor-bearing hosts ↓↓↓CD80, CD86, MHC class II in DCs. ↓↓↓IL-12 expression in DCs ↓↓↓IL-1β, IL-6, and TNFα in DCs ↓↓↓Metastasis ↓↓↓NFĸB signaling	Breast, colon, colorectal, head and neck, bladder, skin, ovarian pancreatic, and prostate cancers	In vivo (female athymic nude mice) In vitro (cell lines MDA-MB-435, CCL23, CAL27, UM-SCC1, UM-SCCC14A)	[138,167,168]
*Tinospora cordifolia*	↑↑↑T- and B-lymphocyte proliferation ↑↑↑T-lymphocytes subsets (CD4+ and CD8+) ↑↑↑Th1 and Th2 cytokine secretion	Oral squamous carcinoma, colon, and cervical cancers	In vivo (male Wistar Kyoto rats) In vitro (cell lines KB, CHOK-1, HT-29, SiHa and murine primary cells)	[169]
Apigenin	↑↑↑IFN-γ-induced activation of STAT1 ↑↑↑T-cell immunity ↑↑↑Sensitive to T cell-mediated cell death ↑↑↑CD4+CD8+ T-cells ↓↓↓PD-L1 in DCs ↓↓↓Tregs ↓↓↓Tumor weights and splenomegaly stabilized Ikaros expression in vitro and in vivo by targeting CK2	Melanoma, colorectal, breast, lung, prostate, leukemia, ovarian cancers	In vivo (C57BL/6 mice) In vitro (cell lines A375, A2058, RPMI-7951, Jurkat cells)	[170,171]
Carotenoids	↑↑↑B- and T-lymphocyte proliferation ↑↑↑Macrophage activity ↑↑↑Cytotoxic T-cells and effector T-cell function ↑↑↑Cytokines	Breast, cervical, ovarian, and Colorectal cancers	In vivo (SJL/J mice)	[172,173]
β-carotene	↑↑↑CD4^+^ T-cell ↑↑↑NK cells ↑↑↑Cells with markers for IL-2 activation ↑↑NK cell cytotoxicity and total T-cells	Gastric, cervical, prostate, breast, colon cancers, and leukemia	In vivo (SJL/J mice)	[169,174]
Lycopene	↑↑Blood IL-2, IL-4, IL-10, TNF-α levels ↑↑Blood IgA, IgG and IgM levels ↓↓↓IL-6	Prostate, breast, and lung cancers	In vivo (female Wistar rats) In vitro (cell lines MCF-10a, MCF-7, MDA-MB-231, HBL-100)	[6]
β-carotene and Lycopene	↑↑CD3+, CD4+, CD8+ cells ↑↑β cells and T-helper cells (CD4+ total cell numbers) ↑↑ IgG	Breast adenocarcinoma	In vivo (SJL/J mice)	[175]
Flavonoids (chalcones, flavones, isoflavones, flavanones, flavanols, anthocyanins)	↑↑ T regulatory subset ↓↓↓ mTOR activity	Breast, stomach, and lung cancers	In vivo (SJL/J mice)	[6]
Luteolin	↑↑↑COX-2 ↓↓↓Total cell, neutrophil, eosinophil counts ↓↓↓IL-4 ↓↓↓IFN-γ ↓↓↓ TNF-α ↓↓↓ T-cell proliferation and antigen-specific ↓↓↓ Mast cell histamine secretion	Breast cancer	In vivo (C57BL/6 mice) In vitro (cell lines TC-1, B16, B16E7)	[6]
Epigallocatechin-3-Gallate	↑↑CD8^+^ and CD4^+^ T cell-mediated immune responses	Head and neck, breast, prostate, stomach, esophagus, colon, pancreas, skin, lung cancers		[176]

The upwards arrows indicate upregulation and the downwards arrows indicate downregulation.

**Table 5:** Ongoing clinical trials on phytochemical efficacy in cancer treatment.

Study Type	Phtochemical (s)	Cancer	Refs.
Study with Human Participants	*Allium sativum*	Colorectal, liver, pancreatic cancer	[212,213]
		Colon adenoma	
Phase I Clinical Trail	Curcumin alone, curcumin + quercetin	Pancreatic cancer	[188]
		Oral leukoplakia	[189]
		Cervical intraepithelial neo-plasia	[188]
		Multiple myeloma	[189]
		Advanced colorectal cancer	[189]
Phase II Clinical Trial	Curcumin	Aberrant crypt foci	[161]
Study with Human Participants	Resveratrol	Reduced insulin-like growth factor (IGF)-1 and IGF-binding protein 3 in the plasma, inhibited tumor formation, and metastasis. Decreased cytochrome P450 and CYP3A4 levels, and increased CYP1A2 in the plasma of healthy person	[172]
Phase II Clinical Trail	Green Tea	Prostate cancer	[198]
Study with Human Participants	β-carotene, α-tocopherol, selenium	Gastric cancer	[199]
